# Effects of Temperature and Pressure of Hot Isostatic Pressing on the Grain Structure of Powder Metallurgy Superalloy

**DOI:** 10.3390/ma11020328

**Published:** 2018-02-24

**Authors:** Liming Tan, Guoai He, Feng Liu, Yunping Li, Liang Jiang

**Affiliations:** 1 State Key Laboratory of Powder Metallurgy, Central South University, Changsha 410083, China; limingtan@csu.edu.cn (L.T.); lyping@csu.edu.cn (Y.L.); liang.jiang@csu.edu.cn (L.J.); 2 Powder Metallurgy Research Institute, Central South University, Changsha 410083, China; 3 High Temperature Materials Research Institute, Central South University, Changsha 410083, China; 4 State Key Laboratory of High Performance Complex Manufacturing, Central South University, Changsha 410083, China; heguoai@csu.edu.cn; 5 Light Alloy Research Institute, Central South University, Changsha 410083, China

**Keywords:** powder metallurgy superalloy, hot isostatic pressing, prior particle boundary, grain structure

## Abstract

The microstructure with homogeneously distributed grains and less prior particle boundary (PPB) precipitates is always desired for powder metallurgy superalloys after hot isostatic pressing (HIPping). In this work, we studied the effects of HIPping parameters, temperature and pressure on the grain structure in PM superalloy FGH96, by means of scanning electron microscope (SEM), electron backscatter diffraction (EBSD), transmission electron microscope (TEM) and Time-of-flight secondary ion spectrometry (ToF-SIMS). It was found that temperature and pressure played different roles in controlling PPB precipitation and grain structure during HIPping, the tendency of grain coarsening under high temperature could be inhibited by increasing HIPping pressure which facilitates the recrystallization. In general, relatively high temperature and pressure of HIPping were preferred to obtain an as-HIPped superalloy FGH96 with diminished PPB precipitation and homogeneously refined grains.

## 1. Introduction

Nickel-based polycrystalline superalloys are widely used as high temperature materials for turbine discs of advanced aircraft engines, owing to their excellent mechanical properties at elevated temperatures [1,2,3,4]. For polycrystalline superalloys with complex compositions, powder metallurgy is an essential manufacturing route, since it yields microstructure with less elemental macrosegregation [3,5]. Hot isostatic pressing (HIPping) is extensively adopted as a methodology of powder consolidation, which has attracted sustainable attention over the last three decades [6,7,8,9]. In a conventional and costly powder route, the hot processes, such as hot extrusion and isothermal forging after HIPping, are mostly utilized to obtain disc parts with a desired microstructure [10]. Direct hot isostatic pressing (as-HIP) could dramatically decrease the cost of PM superalloys, and it is capable of producing near-net or net shape parts [8,11,12,13,14].

However, an unexpected phenomenon, the prior particle boundary (PPB) precipitation, occurs during HIPping and leads to lower ductility and inferior stress rupture properties, which limits the further development of as-HIP [15,16,17]. The precipitates on PPB are found consisting of oxides, carbides, oxy-carbides, γ′ precipitates, and so on [18,19,20]. Previous works indicated that HIPping at high temperature or subsequent supersolvus heat treatment could reduce the detrimental effects of PPB precipitation by dissolving partial precipitates and impelling grain boundary to migrate beyond the precipitates on PPB [9,16,21]. Nevertheless, HIPping and annealing under relatively high temperature make the grains coarsen and thereby decreases the strength of the final article according to the Hall-Petch relation [9,22,23]. Hence, to obtain homogeneous fine-grain structure with minimized impacts from PPB precipitation, it is critical to understand the interaction of grain structure evolution and PPB precipitates under different processing conditions.

By setting four different HIPping experiments, this work studied the influence of temperature and pressure of HIPping on the microstructure evolution of PM superalloy FGH96. The techniques of time-of-flight secondary ion spectrometry (ToF-SIMS) and high-angle annular detector dark-field scanning transmission electron microscopy (HAADF-STEM) was adopted to characterize the element compositions and distributions of PPB precipitates in PM superalloy. Combined with other results and analysis concerning the interactions among HIPping parameters, precipitates and grain evolution, it illustrated the sensitivity of grain structure to the HIPping parameters, and indicated the potential of as-HIP for PM superalloys.

## 2. Materials and Methods

The nominal composition of PM superalloy FGH96 used in this work is listed in Table 1. In practice, the powder of FGH96 was prepared by argon atomization using atomization equipment HERMIGA 100/20 from Phoenix Scientific Industries (PSI, Hailsham, UK) Limited, then the screened powder below 74 μm was loaded into a steel container and degassed to 10^−3^ Pa at 400 °C, following which the powder was consolidated by HIPping under different conditions as indicated in Table 2. Specifically, the average size of powders ranging from 0 to 74 μm is 42 μm. During HIPping, the temperature and pressure were increased simultaneously, and held at set conditions for 2 h, after that, the billets were gradually cooled within furnace.

To characterize the morphology of γ′ precipitates and PPB after HIPping, scanning electron microscope (SEM) observation was performed under a field emission gun SEM FEI Quanta 650 equipped with an electron backscatter diffraction (EBSD) detector. To observe γ′ phase, the mechanically polished samples were etched in a reagent (33 vol % HNO_3_, 33 vol % acetic acid, 33 vol % H_2_O, 1 vol % HF) for 30 to 60 s. The precipitate size distributions were estimated by using Image Pro Plus software (7.0, Media Cybernetics, Inc., Rockville, MD, USA) and taking the equivalent diameter for each precipitate. Additionally, grain structure in four HIPped superalloys was studied by EBSD, the samples were polished by abrasive papers, followed by vibration polishing for over 8 h. EBSD scan step size for all samples was set as 0.5 μm to guarantee enough pixels in each detected grain. The EBSD data were analyzed via HKL CHANNEL5 software (Oxford Instruments, Hobro, Denmark) and the equivalent grain size was calculated based on the measurement of grain area Area_Grain_, $2\sqrt{\frac{{\mathrm{Area}}_{\mathrm{Grain}}}{\pi }}$.

Transmission electron microscope (TEM) samples were made by slices in diameter of 3 mm cut from corresponding alloys with thickness about 50 μm and twin-jet electropolished in a reagent of 90 vol % ethanol and 10 vol % perchloric acid under −25 °C and 20 V. TEM observation was carried out on FEI TEM instruments Tecnai G2 F20 (Hillsboro, OR, USA) and Titan G2 60–300 (Hillsboro, OR, USA) with accelerating voltage of 200 KV and 300 kV respectively, and the images of high-angle annular detector dark-field scanning transmission electron microscopy (HAADF-STEM) was obtained from Titan G2 60–300 (Hillsboro, OR, USA), which clearly presented the relative location of compounds at PPB. 

Time-of-flight secondary ion spectrometry (ToF-SIMS) installed at FERA3 microscope (Tescan, Brno, Czech Republic) was used to detect the segregation or distribution of alloying elements at PPB in larger scale than HAADF-STEM. The positive Xe ions energy of ToF-SIMS was set as 30 keV and the ion beam current was 50 pA.

## 3. Results

**Figure 1:**
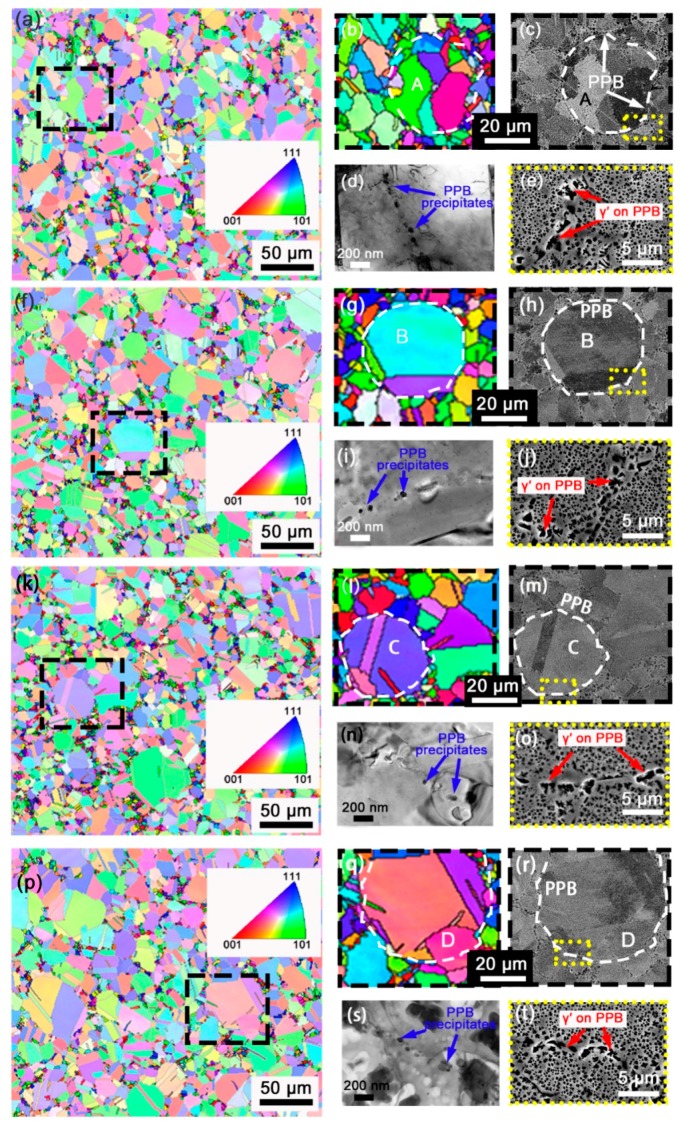
Images showing the microstructure of FGH96 after HIPping under different conditions. (**a**–**e**) 1120 °C/150 MPa; (**f**–**j**) 1170 °C/150 MPa; (**k**–**o**) 1120 °C/120 MPa; (**p**–**t**) 1170 °C/120 MPa; (**a**,**f**,**k**,**p**) EBSD inverse pole figures (IPF) maps highlighting grains with equivalent diameter under 5 μm; (**b**,**g**,**l**,**q**) magnified images of the corresponding areas outlined by dashed lines in black; (**c**,**h**,**m**,**r**) SEM images showing the morphologies of PPB outlined by dashed lines in white; (**d**,**i**,**n**,**s**) TEM images showing the tiny precipitates on PPB; (**e**,**j**,**o**,**t**) SEM images showing the large γ′ on PPB in HIPped alloys after etching.

Figure 1 shows the microstructures of four FGH96 superalloys after HIPping at different conditions from various scales by EBSD, SEM, and TEM. By combining the SEM and EBSD maps at the corresponding locations of PPB, it is found that a large part of PPB acts as grain boundary after HIPping, especially for the PPB with high sphericity which outlines the original shape of the powder with less distortion during HIPping. In some cases, partial PPB is enclosed in grains, such as the grain A and D in HIP96-1 and HIP96-4 shown in Figure 1. A series of small grains are located along PPB as highlighted in the EBSD IPF maps.

The TEM and SEM images demonstrate that a number of small precipitates and irregularly large γ′ existed at PPB. The tiny precipitates below 200 nm are related to oxides and carbides, such as ZrO_2_, TiC, NbC, etc., as described in many other works [20,22,24,25]. The size and distribution of these precipitates on PPB are hardly different in the four specimens at that scale. 

**Figure 2:**
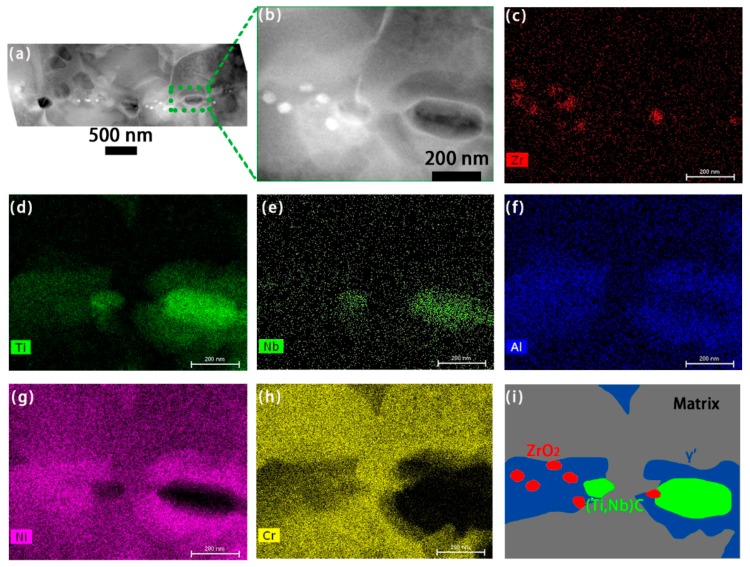
Images showing the elements and compounds at PPB of HIP96-2, in specific, (**a**,**b**) HADDF-STEM images; (**c**–**h**) corresponding EDS mappings of Zr, Ti, Nb, Al, Ni and Cr respectively; (**i**) schematic illustrating relative location of different compounds.

Specifically, HADDF-STEM images and corresponding EDS mappings present more significant information about the size and shape of PPB precipitates, and the element distribution in HIP96-2, as indicated in Figure 2. ZrO_2_ with high concentration of Zr is smaller than the (Ti, Nb)C carbides. The discontinuously distributed compounds on PPB, oxides and carbides, are mainly embedded in large γ′-Ni_3_(Al, Ti) precipitates.

**Figure 3:**
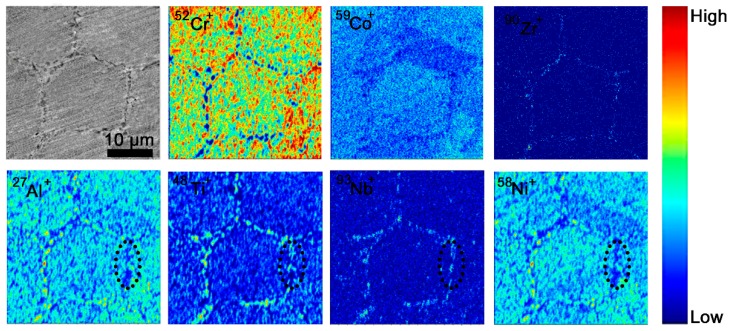
ToF-SIMS results showing the distribution of corresponding detected element ions in HIP96-2 after HIPping, wherein the grey one is the SEM image indicating the presence of PPB and the color maps show element ions detected by ToF-SIMS, the area in dashed line show the segregation of Ti, Nb and impoverishment of Ni, Al. The color bar at right reflects the relative content of detected ions.

As mentioned above, the compounds such as ZrO_2_, (Ti, Nb)C, and γ′ are the detected PPB precipitates in this work, to verify that in larger scale, the ToF-SIMS experiment is performed on FGH96-2. As shown in Figure 3, the ToF-SIMS results indicate that the Zr, Ti, Nb, Al, Ni are segregated at PPB in various degree. Zr corresponds to the ZrO_2_ oxide along PPB. Since Ti, Nb, Al, Ni are γ′-Ni_3_(Al, Ti, Nb) forming elements, and Ti, Nb also tend to form (Ti, Nb)C carbide, it seems hard to identify the existence of these two kinds of compounds by judging the element distribution, but some specific areas of PPB, like the regions outlined by the dashed black lines in Figure 3, are segregated by carbide forming elements Ti, Nb and lack Ni and Al, which proves the presence of (Ti, Nb)C carbide at PPB; the other PPB parts with significant segregation of Ni, Al, Nb and Ti may contain the combination of the abovementioned compounds.

**Figure 4:**
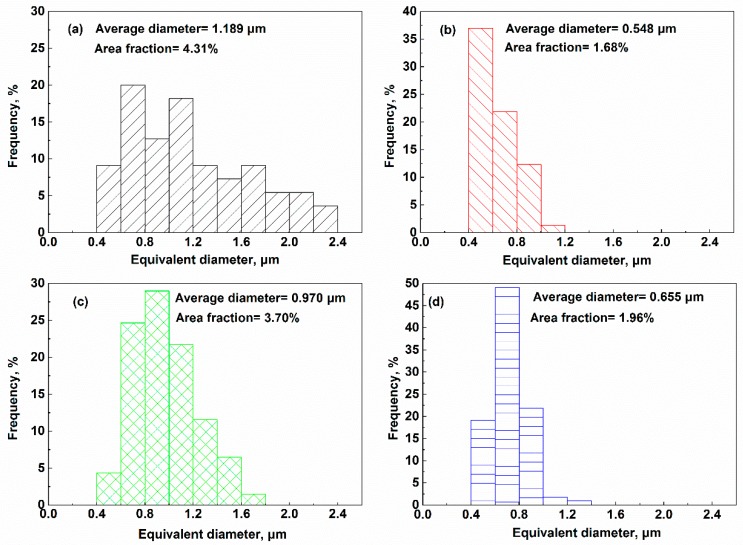
Size distributions of primary γ′ on PPB after HIPping under different conditions. (**a**) 1120 °C/150 MPa; (**b**) 1170 °C/150 MPa; (**c**) 1120 °C/120 MPa; (**d**) 1170 °C/120 MPa.

The size distributions of large primary γ′ at PPB vary from one to another, which are indicated in Figure 4. Generally, γ′ at PPB in alloys HIPped at 1170 °C is smaller. However, the effect of HIPping pressure on the γ′ precipitates seems unclear, the average size of the primary γ′ at PPB drops at near-γ′ solvus HIPping with the pressure decreased, inversely, the average diameter of the γ′ on PPB increases slightly with the decline of the HIPping pressure under higher temperature.

**Figure 5:**
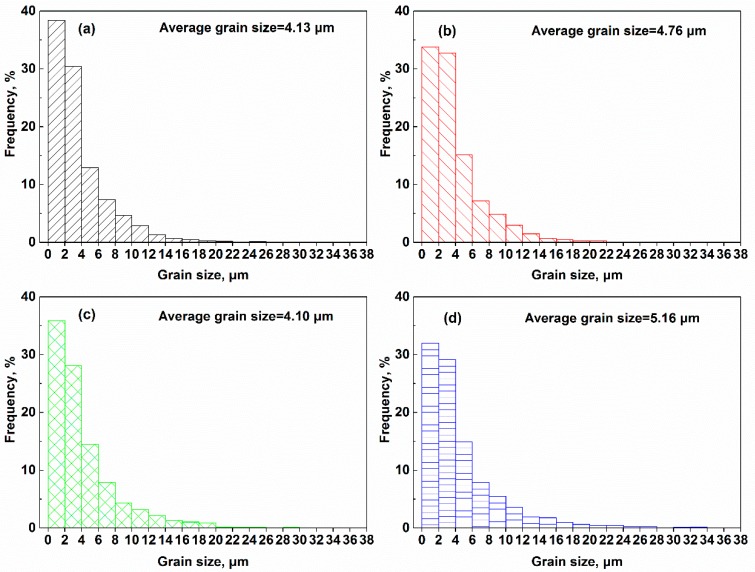
The grain size distributions of four FGH96 superalloys after HIPping under different conditions, (**a**) 1120 °C/150 MPa; (**b**) 1170 °C/150 MPa; (**c**) 1120 °C/120 MPa; (**d**) 1170 °C/120 MPa.

Additionally, the grain size distributions and average grain sizes at different HIPping conditions are various, as shown in Figure 5. Firstly, the temperature has more significant effects on the grain structure, the frequency of small grains decreases as the HIPping temperature changes from near-γ′ solvus to high supersolvus, and the average grain size increases correspondingly. The pressure has no apparent influence on the grain structure in alloys HIPped at 1120 °C; but under supersolvus temperature 1170 °C, the average grain size increases slightly at lower HIPping pressure.

## 4. Discussion

As mentioned previously, the precipitates on PPB can be divided into two types, namely, the small refractory oxide and blocky carbide compounds, and the irregularly large γ′. The oxide and carbide are mainly formed by diffusion and segregation of Zr, Ti, Nb, etc., for example, the Zr could react with residual oxygen in the container at extremely low partial oxygen pressure and moderate temperature; some of the oxides may have already formed during atomization prior to consolidation [20,26,27]. The stable MC-type carbide starts to precipitate by element segregation or dissolution of metastable carbide which releases the forming elements of the carbide [24,28]. The oxide and carbide precipitate at the early stage of HIPping in a rapid speed and remain highly stable at elevated temperatures, thereby the morphology and distribution of this type of compound under four sets of HIPping parameters has less difference at the scale of TEM. 

On the other hand, the γ′ on PPB is dramatically impacted by the temperature of HIPping, as γ′ on PPB starts to dissolute into matrix when the HIPing temperature exceeds γ′ solvus. Since the dissolution of γ′ is a diffusion-controlled process, the diffusion coefficient of solute can basically reflect its speed. As shown in Equation (1), the diffusion coefficient of solute, D, is closely related with temperature,

(1) $$D=D_{0}\exp(\frac{-Q}{RT})$$

in which the *Q* represents diffusion activity energy, *D*_0_ is deemed as a constant. Since dissolution is diffusion controlled, the higher supersolvus temperature of HIPing facilitates the process, and the primary γ′ dissolves into matrix rapidly as the HIPing temperature reaches 1170 °C, which causes the dramatic decrease of its average size and fraction.

The temperature and pressure could directly modify the grain structure through affecting mechanisms of recovery (RV), recrystallization (RX) and grain growth. With the temperature increased, the yield strength of powders declines and the powder starts to deform which introduces accumulated dislocations in powders [17]. Generally, to initial RX, critical dislocation density should be satisfied, and the dislocation density in the bulk ρ under isothermal deformation is related with RV and working hardening before recrystallization as described by Equations (2) and (3) [29,30]:

(2) $$\frac{d\rho }{d\varepsilon }=\frac{d\rho^{+}}{d\varepsilon }-\frac{d\rho^{-}}{d\varepsilon }$$

(3) $$\frac{d\rho }{dt}{=k}_{1}\sqrt{\rho }-k_{2}\rho$$

wherein, t is the time, *ε* represents equivalent strain. The right two positive terms of above equations correspond to work hardening and RV respectively. The work hardening term, $\frac{d\rho^{+}}{d\varepsilon }$, which equals to $k_{1}\sqrt{\rho }$, means that the accumulation of moving dislocations varies with imposed strain, k_1_ means the storage coefficient; the recovery term, $\frac{d\rho^{-}}{d\varepsilon }$, equivalent to k_2_*ρ*, indicates that dislocation annihilation is enhanced with increasing of strain. k_2_ depends essentially on temperature, as RV is a thermal activated process which mainly dominated by glide or climb of dislocations, hence higher temperature facilitates RV and the decrease of dislocation density [31]. On the other hand, higher pressure yields higher strain energy in the matter and accelerate the RX, which contributes to the drop of grain size after HIPping. 

In terms of grain growth, the grain boundary migration velocity *v* can be expressed by Equations (4) and (5) [30,32]:

(4) v=M·ΔP

(5) $$\Delta P=P_{D}-P_{R}$$

wherein the driving force per unit volume of boundary ΔP derives from the difference between the thermodynamic driving pressure *P*_D_ and the resistive pressure *P_R_*. *M* is the mobility of the grain boundary, which can be estimated by [30,33]:

(6) $$M=M_{0}\cdot \exp(\frac{-Q_{app}}{RT})$$

where the *M*_0_ is the pre-exponential factor, and positive *Q_app_* is the apparent activation energy. By combining Equations (4) and (6), we can see that increasing HIPping temperature accelerates grain growth. 

Additionally, the interaction of moving grain boundary and secondary phase particles is hardly ignorable considering the Zener pining effect contributing to P_R_, the resistance of second-phase particle on the moving dislocation or grain boundary [34,35]. The general Zener pining effect P_*Z*_ is expressed as [36]:

(7) $$P_{Z}=\frac{3V_{f}\cdot \gamma }{2r}$$

where, the *r* is the radius of particle size, *γ* is the grain boundary energy, and *V_f_* is the volume fraction of particle. 

As concluded by Song and Aindow [37], comparing with oxide and carbide precipitates, the γ′ on PPB with much larger volume fraction plays a dominating role on restraining grain boundary migration. In addition, the less precipitates are mainly surrounded by the primary γ′ since they are preferential nucleation sites of γ′ [20,32], which further weakens their pining effects. Equation (7) reflects that the difference of the pining force of γ′ at PPB is generated from the ratios of V_f_/r. Assuming the area fraction of γ′ at PPB V_a_ equals to *V_f_*, the ratios *V_f_*/*r* of four HIPped alloys HIP96-1, HIP96-2, HIP96-3, HIP96-4 are estimated to be 0.036, 0.031, 0.038 and 0.030 μm^−1^, respectively, illustrating that the pining force in the FGH96 alloys HIPped under higher temperature is weaker. 

In this work, higher temperature of HIPping increases the mobility of the grain boundary, and contributes to the reduction of pining force from γ′ precipitate at PPB. Higher pressure facilitates the deformation and RX of powders under consolidation, which contributes to the drop of grain size after HIPping.

## 5. Conclusions

The PPB precipitation and grain structure in nickel-based superalloy FGH96 after HIPping under different temperature and pressure were studied in this work. From the above results and analysis, the following conclusions can be reached:

(1) The PPB precipitates mainly consisted of large primary γ′, small ZrO_2_ oxides and MC carbides in the four HIPped FGH96 superalloys.
(2) Comparing with HIPping under near-γ′ solvus 1120 °C, HIPping at 1170 °C accelerates the grain boundary migration and dissolution of γ′ on PPB, which impels the moving grain boundary to bypass the PPB.
(3) As RX is facilitated under higher pressure, HIPping at 150 MPa is preferred to obtain a microstructure with homogeneously refined grains.
(4) In general, reducing the pining effects by dissolution of PPB precipitates through HIPping at high supersolvus temperature may induce excessive grain growth of grains, but increasing the pressure could suppress this tendency.

## Figures and Tables

**Table 1:** Nominal composition of FGH96 in wt %.

Co	Cr	Mo	W	Al	Ti	Nb	B	Zr	C	Ni
13.0	16.0	4.0	4.0	2.1	3.7	0.7	0.015	0.03	0.03	Bal.

**Table 2:** HIPping parameters of four samples.

Samples	Temperature °C	Pressure MPa
HIP96-1	1120	150
HIP96-2	1170	150
HIP96-3	1120	120
HIP96-4	1170	120
